# An Inaugural Dissertation on Intra-Stomatic Galvanism, Submitted to the Trustees and Faculty of the Ohio College of Dental Surgery, for the Degree of Doctor of Dental Surgery, February 10, 1854

**Published:** 1854-04

**Authors:** George Watt

**Affiliations:** Xenia, Ohio


### DENTAL REGISTER OF THE WEST.

VOL. VII. APRIL, 1854. No. 3.

### Original Communications.

*An Inaugural Dissertation on Intra-Stomatic Galvanism, submitted to the Trustees and Faculty of the Ohio College of Dental Surgery, for the Degree of Doctor of Dental Surgery, February* 10, 1854. *By* GEORGE WATT, M. D., *Xenia, Ohio.*

WHEN two solid conductors of electricity are plunged into a liquid which acts upon them unequally, the electric equilibrium is disturbed; the one acquiring the positive, the other the negative condition. Zinc and platinum put into dilute sulphuric acid constitute such an arrangement, and electric force is thus generated; the zinc, being the metal attacked, becomes negative, and the platinum remaining unaltered, becomes positive. In the closed circle, however, the relative states of the two metals in the exciting cell are reversed, the zinc being positive and the platinum negative.

With these facts, and with galvanic action in general, the profession are familiar, but many seem to be unaware of their importance in dental surgery.

We do not propose to notice the science of galvanism in general, but will confine our remarks, principally, to its actions and influence within the mouth. But, to arrive at a proper understanding of these, it is necessary to observe some of the elementary facts and leading principles of the science.

The essential elements of a galvanic battery are one imperfect and two perfect conductors, or one perfect and two imperfect conductors. The metals, charcoal and most animal substances, though differing in conducting power, are called perfect, while water and aqueous solutions are called imperfect conductors. In the battery first described, it is essential that the imperfect act chemically on one of the perfect conductors.

The voltaic current is the great instrument of chemical decomposition, especially of binary compounds. Galvanic decomposition is called *electrolysis;* a substance which may be thus decomposed is an *electrolyte*, and the terminations of the battery are called *electrodes*.

One of the indispensable conditions of electrolysis is fluidity. Bodies which, when liquid, conduct freely, and as freely suffer decomposition, are absolute insulators to voltaic electricity in the solid state. All liquids, however, are not electrolytes. Many refuse to conduct, and consequently no decomposition can occur.

It becomes necessary here, to distinguish carefully between primary and secondary decomposition, that is, between true electrolysis and that decomposition brought about by the reaction of the substances eliminated upon the surrounding fluid, or upon the electrodes. These secondary actions are very numerous, the disunited elements being presented in their nascent form which is peculiarly favorable to chemical action. The voltaic decomposition of most acids and salts is to be attributed to these secondary actions.

Electrolytic decomposition depends on the quantity of the electric fluid passing through the electrolyte, but the secondary actions depend on the nature and quantity of the agents evolved by the former. Thus, hydrochloric acid has no effect on gold; but when, by electrolysis, its chlorine is evolved against a gold electrode, chloride of gold is formed.

By means of the magnetic galvanometer, it has been demonstrated that the quantity of electricity traversing an acid solution of uniform strength, varies inversely as the square root of the distance between the plates—the distance varying from 4 to 1, the current will be as 1 to 2. Thus it will be seen that very small exciting surfaces may produce vigorous chemical action, when the distance between them is but trifling. Accordingly, the late Professor Fownes remarks: "A pair of small wires of zinc and platinum, dipping into a single drop of dilute acid, developes far more electricity, to judge from the chemical effects of such an arrangement, than very many turns of a large plate electrical machine in high action, yet electrolytic decomposttion can be distinctly and satisfactorily effected by the latter."

In comparing the effects of electric currents, the late Dr. Turner remarks: "Feeble agencies, operative for a long period, are often just as efficacious in effecting great changes as powerful agents, at work during a short period."

Bearing the above principles in mind, let us apply them practically to the mouth.

We do not propose to go into the analysis of the fluids of the mouth, nor, of the foreign substances introduced into it, as this would require space beyond our present limits. Suffice it to say, that the saliva has a strong affinity for oxygen and absorbs it readily from the atmosphere; that it corrodes many of the metals; that it is an electrolyte; and that acidity is generally a result of its decomposition.

Let us now notice some conditions of the mouth artificially produced. It is universally admitted that a tooth surrounded by a clasp is apt to decay, and a variety of reasons are offered to explain this tendency. It is evident that it is often, if not always, promoted by galvanic action. A portion of food, a piece of animal fibre, for instance, lodges between the tooth and clasp, a perfect battery of the first kind, is thus formed, the saliva being the imperfect, and the clasp and fibre the perfect conductors. The other requisite condition is also present, for the saliva acts chemically on the fibre. This being the case, the binary compounds of the saliva are electrolyzed. Water being one of them, of course its oxygen goes to the positive side, combines with the nitrogen of the fibre, and nitric acid is produced, which dissolves the enamel as fast as formed. The enamel being removed, the animal portion of the tooth forms a part of the battery, and the current continues without the lodgement of a foreign substance.

It is evident that the tooth, being a solid substance, is not electrolyzed, but that its carious condition is a result of secondary decomposition. We have shown that it may take place from the electrolysis of the water of the saliva. It may also take place from the decomposition of the chloride of sodium, the chlorine, being evolved at the tooth, will displace the phosphoric acid, and take up the lime of the enamel. It may take place from the electrolysis of almost any of the binary principles of the fluids of the mouth, or of all of them, as they will be decomposed in the ratio of their equivalents.

Galvanic action is often produced in the mouth by the use of metals or alloys which are easily oxydized, and more especially, when two or more are used which differ in their affinity for oxygen. This often results from the use of solder of a low carat, or from silver solder used on gold plate. It *always* takes place when any of the "thousand and one" amalgams are used for filling teeth. Minute circles, similar to those on the surface of excited commercial zinc, are always produced by the action of the saliva on these fillings. The greater the number of metals used, the more energetic will these circles be, and if their affinity for oxygen differs greatly, the most oxydizable will, of course, corrode more rapidly, on account of the currents thus established. When the oxydation progresses till but one metal remains, the porosity of the filling presents a condition peculiarly favorable to secondary decomposition, which, as in the former case, causes the destruction of the tooth.

The minute current established by these compound fillings decompose the binary compounds of the saliva, just as those of excited commercial zinc decompose the water of the exciting solution. To understand one obvious effect of secondary action in these cases, we have only to reflect that the chlorine of the chloride of sodium, and the hydrogen of the water, are set free, unite and form hydrochloric acid, and that all this takes place in contact with the tooth. When approximal cavities are filled with these "compounds," the adjacent teeth are often thus destroyed.

When the adjoining tooth is filled with gold, we have a most powerful battery of the first kind. We have often seen an amalgam filling which had remained in the mouth without serious results, produce all its deleterious effects, on the introduction of a gold filling into a neighboring tooth. Since we began writing this, we saw a case which illustrates our position: In a cavity on the labial surface of the second lower molar, was a gold filling, and an amalgam plug occupied the corresponding position in the dens sapientiæ. We will be minute here, for we wish to be understood. Let the gold represent the copper, and the amalgam the zinc of the ordinary apparatus Both being, as it were, in the exciting cell, the gold is negative, and the amalgam positive. Now, in their electrolytic action, all the binary compounds of the saliva, as already remarked, are decomposed, and the electro-negative elements, of course, go to the amalgam filling. Of water, the oxygen, and of chlorine of sodium, the chlorine appear there, and, being in their nascent state, unite and form chloric acid. Nothing but the destruction or the tooth containing the amalgam, could be expected under such circumstances; and it was, accordingly, found in a softened condition. This is one case—we have seen more than a hundred similar ones.

It will be observed, that we have said nothing concerning the deleterious effects of these amalgams on the general constitution. Nothing is needed, for "TEKEL" is stamped upon them. We have intentionally confined our remarks to their influence on the teeth alone. In speaking of their constitutional effects, Professor Piggot justly remarks: "The amalgam question, as it has been called, is thus answered with the utmost promptitude by chemistry. To the chemist it has but one side; it needs but to be stated to be immediately decided upon." We will merely add, in this connection, that chemistry not only answers "with the utmost promptitude," but, from all its departments, gives the same answer, the severest *tortures* failing to elicit any other.

To notice all the sources and results of galvanic action within the mouth, would lead us beyond our present limits. We have spoken of the more frequent causes, and the most obvious effects of these currents. We have throughout supposed the fluids of the mouth to be natural and healthy. If we have demonstrated, by way of illustration, that three of the strongest acids known to chemistry, are, in their nascent state, brought in contact with the teeth, we have at least partially succeeded in our undertaking.

